# Polysaccharides Extracted From *Panax* Ginseng C.A. Mey Enhance Complement Component 4 Biosynthesis in Human Hepatocytes

**DOI:** 10.3389/fphar.2021.734394

**Published:** 2021-09-10

**Authors:** Shuang Liu, Fangbing Liu, Tingting Wang, Jianzeng Liu, Cheng Hu, Liwei Sun, Guan Wang

**Affiliations:** ^1^ National Engineering Laboratory for AIDS Vaccine, Key Laboratory for Molecular Enzymology and Engineering, School of Life Sciences, Jilin University, Changchun, China; ^2^ Jilin Ginseng Academy, Changchun University of Chinese Medicine, Changchun, China; ^3^ Research Center of Traditional Chinese Medicine, The Affiliated Hospital of Changchun University of Chinese Medicine, Key Laboratory of Active Substances and Biological Mechanisms of Ginseng Efficacy, Changchun University of Chinese Medicine, Changchun, China

**Keywords:** ginseng, water-soluble ginseng polysaccharides, complement component 4, C4 transcription, C4 promoter

## Abstract

*Panax* ginseng C.A. Mey (ginseng) is a classic medicinal plant which is well known for enhancing immune capacity. Polysaccharides are one of the main active components of ginseng. We isolated water-soluble ginseng polysaccharides (WGP) and analyzed the physicochemical properties of WGP including molecular weight, monosaccharide composition, and structural characteristics. WGP had minimal effect on the growth of hepatocytes. Interestingly, WGP significantly increased the mRNA and protein levels of complement component 4 (C4), one of the core components of the complement system. Promoter reporter gene assays revealed that WGP significantly enhanced activity of the *C4* gene promoter. Deletion analyses determined that the E-box1 and Sp1 regions play key roles in WGP-induced *C4* transcription. Taken together, our results suggest that WGP promotes C4 biosynthesis through upregulation of transcription. These results provide new explanation for the intrinsic mechanism by which ginseng boosts human immune capacity.

## Introduction

For *Pana*x ginseng C.A. Mey (ginseng), a perennial plant belonging to genus Panax, is one of the most appreciated medicinal plants (Yun, 2001). Regulation of the immune response is one of the main biological activities of ginseng extracts (Wang et al., 2001; Senchina et al., 2009; Kang and Min, 2012; Kachur and Suntres, 2016; Yun and Yi, 2020). The reason for long-lasting usage of ginseng is that it contains numerous natural regulatory compounds, such as polysaccharides, ginsenoside, phytosterols, and peptides (Dai et al., 2017).

As an important active component of ginseng, water-soluble ginseng polysaccharides (WGP) have been proved playing an important role in the modulation of immunity (Lim et al., 2004; Kim et al., 2007; Sun, 2011; Yu et al., 2017; Li et al., 2019). Human immune system is an elaborate and layered defense system against infections through gradual increase of specificity to invading organisms (Medzhitov, 2007). Among the components of immune response, the complement system plays an important role in organismal defense. Complements can lower the B-cell activating threshold and promote antigen retention on the surface of dendritic cells (Janssen et al., 2006; Gros et al., 2008; Carter and Fearon, 2010; Carroll and Isenman, 2012). Interaction between the effector components and the complement receptors can alter the secretion of cytokines and regulate the direction of T cell differentiation by influencing the microenvironment, thereby affecting the outcome of inflammation (Lalli et al., 2008; Amsen et al., 2009; Kolev et al., 2013).

The *C4* gene product, fourth component of human complement (C4), is an important component of the complement system (Galibert et al., 1997). C4 together with C2 forms the classical complement which activates C3 convertase (Dodds and Law, 1990). C4 is an important effector for both innate and adaptive immune systems among vertebrate animals. C4 is mainly expressed in the liver and induced during acute inflammation or tissue injury (Zhang et al., 2009).

Until now, little is known about the effects of ginseng on the production of complement components. In this study, we investigated the effect of WGP on C4 biosynthesis and explored the underlying molecular mechanism. We demonstrate that WGP enhances C4 production by promoting *C4* gene transcription via the E-box1 and Sp1 regions in the promoter. Our results provide more clues to fully understand the mechanism by which ginseng enhances human immunity.

## Materials and Methods

### Preparation of Water-Soluble Ginseng Polysaccharides

Ginseng powder (400 g) was boiled in deionized water (4 L) for 3 h. After filtration, the solid phase was boiled in deionized water at the same ratio (g/ml) two more times. The filtrate was combined, centrifuged, and concentrated. The concentrated solution was mixed with anhydrous ethanol at a volume ratio of 1:3 and left overnight at 4°C. Discard the supernatant by centrifugation and the precipitate was successively washed with 75% ethanol, 95% ethanol, anhydrous ethanol, and ether. The crude ginseng polysaccharides were obtained after drying and subsequently redissolved in deionized water. After removal of proteins using a Sevage reagent, a 3 KD molecular sieve was used to remove small molecules to obtain WGP. The content of sugar was tracked and monitored by the phenol sulfuric acid method (Wang et al., 2020).

### Molecular Weight Analysis of Water-Soluble Ginseng Polysaccharides

WGP (5 mg/ml) was filtered *via* 0.45 μm microfiltration membrane. The filtered sample (20 μL) was loaded into a TSK-Gel G4000PWXL column (Tosoh, Shanghai branch, China) controlled by LC-10Avp system (Shimadzu, Shanghai branch, China). High performance gel permeation chromatography (HPGPC) was performed using 0.2 M NaCl as mobile phase at flow rate of 0.5 ml/min. T-series Dextran standards were used for reference standards.

### Monosaccharide Composition Analysis of Water-Soluble Ginseng Polysaccharides

Monosaccharide composition analysis was performed as previously described (Wang et al., 2020). Briefly, WGP (200 g) was hydrolysed in anhydrous methanol solution (1 ml) containing hydrochloric acid in nitrogen. Then, the sample was dried and hydrolysed in 2 M trifluoroacetate acid. After dried, the sample was dissolved using 0.3 M sodium hydroxide and added an equal volume of 0.5 M PMP (1-phenyl-3-methyl-5-pyrazolone) with thoroughly blending using pipettor. Placed the mixture (0.2 ml) for 30 min at 70°C, added 0.1 ml hydrochloric acid and 0.7 ml dichloromethane for extraction. The aqueous phase was filtered *via* 0.22 μm organic membrane. Conversion of monosaccharides with PMP were detected *via* high-performance liquid chromatography (HPLC). The sample (10 μL) was injected into a 4.6 mm × 250 mm COSMOSIL 5C18-PAQ column (Nacalai Tesque, Shanghai branch, China) controlled by an LC-20AT system (Shimadzu, Shanghai branch, China). HPLC was performed using a mobile phase composed of 19.5% Acetonitrile and 80.5% 0.1 M PBS (pH 7.0) at flow rate of 1 ml/min. The absorbance values at wavelength 245 nm were compared with those of monosaccharide standards including arabinose, fucose, galactose, galacturonic acid, glucose, glucuronic acid, mannose, rhamnose, and xylose to determine the monosaccharide composition of WGP.

### Fourier Transform-Infrared Spectroscopy Analysis

Fourier transform-infrared spectroscopy (FT-IR) spectra of WGP was acquired *via* Tenor 27 spectrophotometer (Shimadzu, Shanghai branch, China). WGP was ground with KBr powder at a mass ratio of 3:1 and compressed into a pellet. The FT-IR spectra were recorded in range of 400–4,000 cm^−1^.

### Nuclear Magnetic Resonance Analysis

The lyophilized WGP was dissolved in D_2_O. The ^1^H and ^13^C nuclear magnetic resonance (NMR) spectrum were performed on AV-500, 600, and 800 instruments (Bruker, Germany) using tetramethylsilane as the internal standard.

### Cell Culture

Human hepatic cell line L-O2 was purchased from the American Type Culture Collection (Manassas, VA, United States). Cells were cultured using DMEM media containing 100 U/ml penicillin, 100 μg/ml streptomycin, 2 mM L-glutamine (Thermo Fisher Scientific, Shanghai branch, China) and 10% (V/V) fetal bovine serum (Abwbio, Guangzhou, China) and incubated in HERAcell150i incubator (Thermo Fisher Scientific) set to 5%CO2/95% air and 37°C. Cells were tested for *mycoplasma* monthly by the PCR method described by Uphoff and Drexler (Uphoff and Drexler, 2005).

### *In Vitro* Cytotoxicity Assays

The L-O2 cells were treated with a series of equal ratio gradient concentrations of WGP for 72 h in a 96-well plate. MTT [3-(4, 5-dimethyl-thiazol-2yl)-2, 5-diphenyl tetrazolium bromide] (Sigma-Aldrich, Shanghai branch, China) was added at the final concentration of 0.5 mg/ml and incubated for 4 h in incubator. Then, the cells were lysed in 10% SDS containing 10 mM HCl overnight. The absorbance values at wavelength 590 nm were measured *via* a microplate reader.

### Western Blotting

Whole cell lysates were obtained *via* ultrasonic cell disruption and subjected to SDS-polyacrylamide gel electrophoresis. Proteins were electrophoretically transferred onto PVDF (polyvinylidene difluoride) membrane (Thermo Fisher Scientific), and subsequently immunoblotted utilizing anti-β-actin (Proteintech, Rosemont, IL, United States) and -C4 (Abcam, Cambridge, MA, United States) antibodies. Visualization of immunoreactive proteins was conducted using the Odyssey Infrared Imaging System (LiCor, Lincoln, NE, United States). Densitometry measurements were performed using Odyssey V3.0 software (LiCor).

### Real-Time PCR

Total RNA was extracted by TRIZOL method and used to make cDNAs *via* reverse transcription PCR Kit (Thermo Fisher Scientific), as described previously (Edwards et al., 2009). The LightCycler 480 real-time PCR meter (Roche, Indianapolis, IN, United States) and TaqMan probe Hs00246758_m1 (Thermo Fisher Scientific) were used to quantify *C4* transcripts. Comparative Ct method was used to calculate the fold changes (Livak and Schmittgen, 2001). *C4* transcripts were normalized to GAPDH transcripts measured by TaqMan probe (Hs02786624_g1).

### Construction of C4 Promoter-Luciferase Reporter Plasmids

The C4 promoter region was PCR amplified from human genomic DNA *via* full length-forward and reverse primers (Table 1). Then, the PCR product was cloned into pGEM-T-Easy vector (Promega, Madison, WI, United States). A single clone of *C4* promoter was identified and digested by XhoI and HindIII (Promega). The *C4* promoter was then subcloned into reporter gene vector pGL4.19 basic (Promega) at the XhoI and HindIII restriction sites to generate pC4-1,007/+44. To generate the 5’-deletion constructs, pC4-119/+44, pC4-102/+44, pC4-92/+44, pC4-72/+44, and pC4-48/+44, genomic DNA fragments were PCR amplified from the pGL4.19 plasmid using forward (segment 1 to segment 5) and reverse primers (Table 1). Then, the PCR product was digested by HindIII and XhoI, and subcloned into pGL4.19 basic.

**TABLE 1:** Primers used in *C4* promoter luciferase assay (5’—3’).

Full length-forward	CCC​TCG​AGA​GAT​TCT​GCT​CAT​CAT​TGC​TCA​GC
Segment 1- forward	CCC​TCG​AGC​CAC​AAC​TCT​GGG​CCT​GA
Segment 2- forward	CCC​TCG​AGA​GGC​CAG​TTG​CAC​TTC​TTG​G
Segment 3- forward	CCC​TCG​AGC​ACT​TCT​TGG​CTG​TCA​CGT​G
Segment 4- forward	CCC​TCG​AGG​TTT​CCC​AGC​TTA​GCT​GG
Segment 5- forward	CCC​TCG​AGG​GAG​GAG​CAA​GGT​CCA​GAG​T
Reverse	GGA​AGC​TTG​GAT​CCA​AGA​GAG​GTT​AGA​TCC

### Statistical Analysis

Statistical analyses were performed using GraphPad Prism (GraphPad Software, San Diego, CA, United States). Differences were compared *via* the non-pair-wised two-sample *t*-test.

## Results

### Isolation and Physicochemical Characterization of Water-Soluble Ginseng Polysaccharides

**FIGURE 1:**
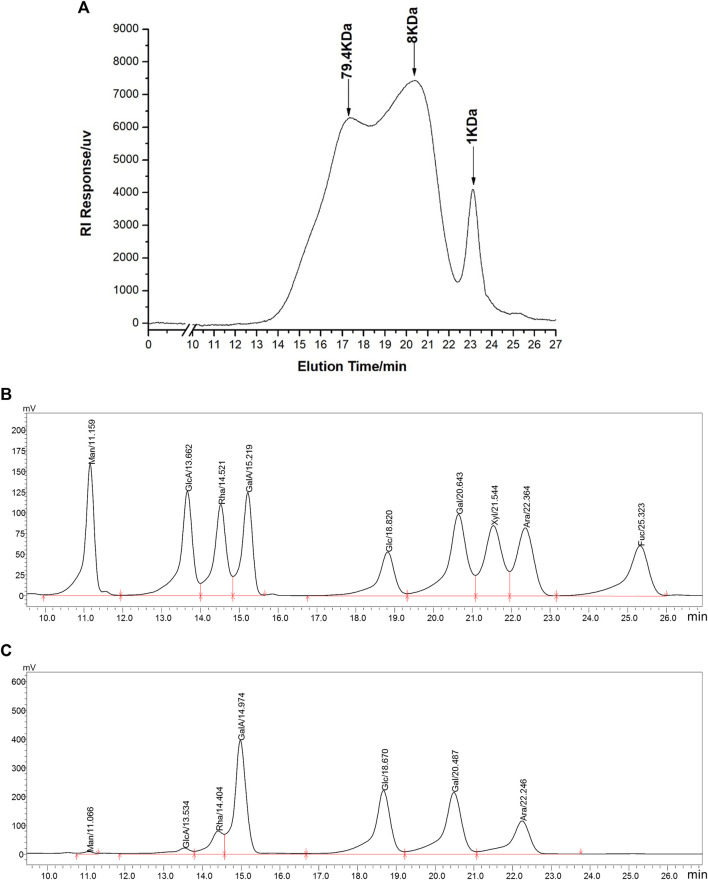
Chromatographic analysis of WGP. HPGPC was used to determine WGP molecular weight distribution **(A)**. HPLC was used to separate monosaccharide standards **(B)** and analysis the monosaccharides composition in WGP **(C)**. Man, GlcA, Rha, GalA, Glc, Gal, Xyl, Ara, and Fuc represent mannose, glucuronic acid, rhamnose, galacturonic acid, glucose, galactose, xylose, arabinose, and fucose, respectively.

WGP were obtained *via* hot-water extraction and alcohol precipitation from ginseng powder. The yield of WGP was 0.85%. Carbohydrate content of WGP was over 98% and protein impurity was less than 0.1%. HPGPC was used for molecular weight analysis of WGP. We obtained two overlapping peaks and a single symmetric peak indicating that the molecular weight range of the WGP was from 1 kD to 79.4 kD (Figure 1A). The peak at 1 kD is due to incomplete interception of molecular sieve. Then, monosaccharide compositions analysis of WGP hydrolysate was performed using HPLC and nine monosaccharide reference standards. Monosaccharide profile was obtained by comparing retention times of nine standards under the same analytical conditions (Figures 1B,C). Chromatographic results demonstrated that WGP was composed of galacturonic acid, galactose, glucose, arabinose, rhamnose, glucuronic acid, and mannose in molar proportions of 28.9: 24.4: 23.0: 13.9: 6.7: 2.6: 0.6, respectively. Xylose and fucose were not detected. Consistent with published studies, glucose, galacturonic acid, and galactose are the most common monosaccharide compositions of WGP (Shi et al., 2017; Shin et al., 2017; Chen et al., 2018; Song et al., 2018; Zhao et al., 2019; Park et al., 2020). However, the specific proportion of these monosaccharides has varied widely across these studies, which may be due to the different origins of ginseng.

**FIGURE 2:**
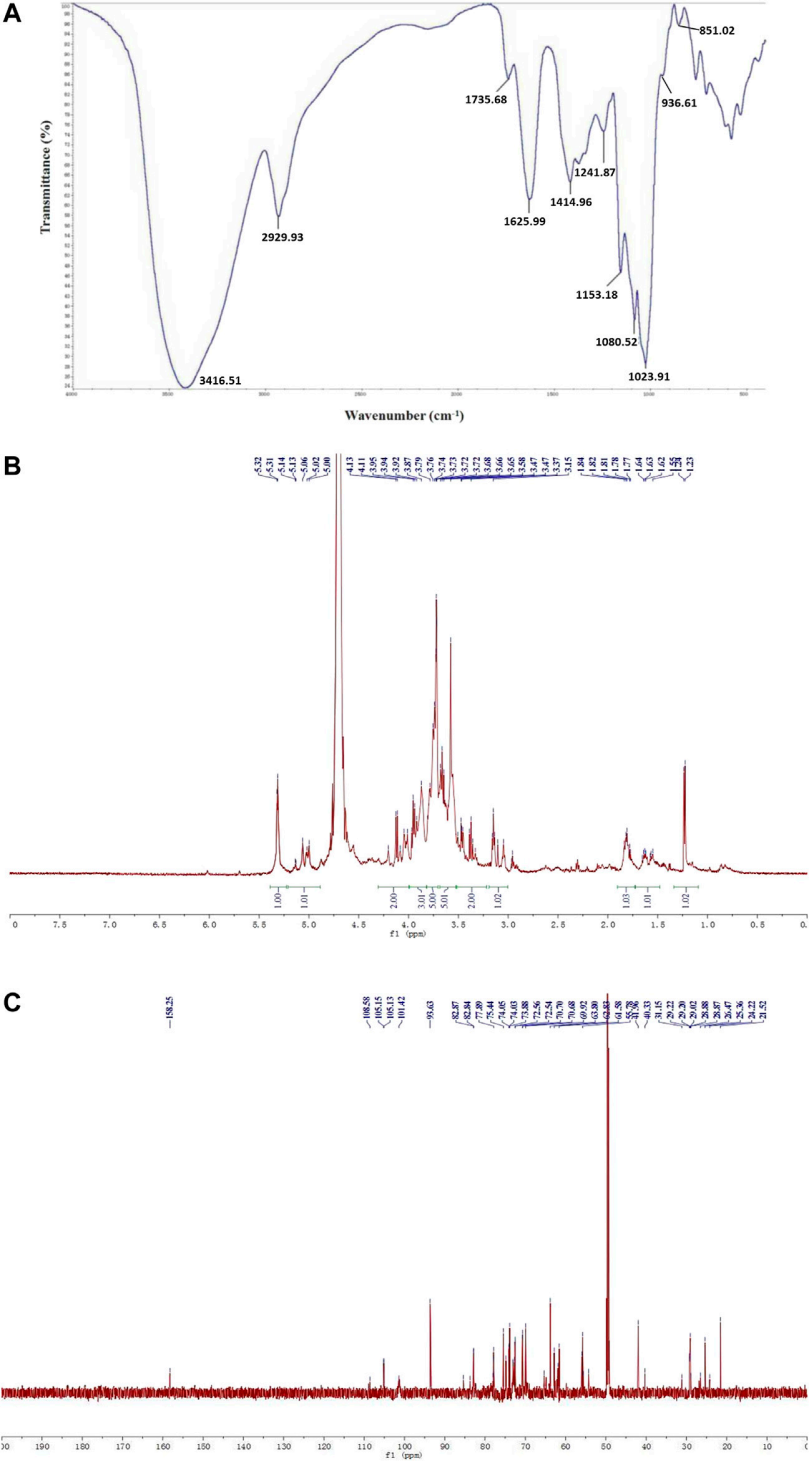
Structural characterization of WGP. FT-IR spectrum with the range of 4,000–400 cm^−1^ **(A)** and ^1^H NMR **(B)**, ^13^C NMR **(C)** spectra were used to characterize WGP structure.

The FT-IR spectrum of WGP showed characteristic hydroxyl and C-H stretching vibration peak at 3,416.51 and 2,929.93 cm^−1^, respectively. The absorption peaks at 1,625.99 and 1735.68 cm^−1^ were caused by bound water and C=O stretching vibration of uronic acid, respectively. The absorption peaks at 1,414.96 and 1,241.87 cm^−1^ represented C-H angular vibrations of carbohydrate. In addition, 1,153.18, 1,080.52, and 1,023.91 cm^−1^ were assigned to C-O-H and C-O-C stretching vibration peaks of pyran, indicated that WGP was connected by α-pyranoside bond. The absorption peaks at 936.61 and 851.02 cm^−1^ were the characteristic absorption peaks of α-Glc*p* (Figure 2A).

WGP ^1^H NMR spectrum exhibited seven anomeric proton signals at δ5.32, 5.31, 5.14, 5.13, 5.06, 5.02, and 5.00 ppm, suggesting that the analyte was made up of seven monosaccharides. Intense signals within δ3.10–4.20 ppm represented CH-O and CH_2_-O groups of carbohydrate. The chemical shift from δ3.15–4.13 ppm was contributed by H-2 to H-6 protons. Meanwhile, no signal was observed at δ5.50 ppm indicating that WGP contained glucopyranose, consistent with the FT-IR result (Figure 2B). The structure of WGP was further analyzed by ^13^C NMR spectroscopy. *β*-1,4-Linked Gal residues exhibited six signals at δ105.15, 70.70, 73.05, 75.44, 74.05, and 61.58 ppm, corresponding to their C-1 to C-6. Signals at δ105.13 and 82.84 ppm were attributed to C-1 and C-3 of *β*-1,3-Gal, respectively. Furthermore, δ101.42 ppm was the heterocephalic carbon position of *α*-Glc*p*. Peak at δ78.04 ppm indicated that C-4 of *α*-Glc*p* had been replaced. δ73.07, 74.81, 72.54, and 62.83 ppm were the positions of C-2, C-3, C-5, and C-6 of *α*-1,4-Glc*p*, respectively*.* The anomeric signals at δ108.58 and 83.72 ppm were due to C-1 and C-3 carbons of *α*-1,3,5-Ara (Figure 2C). These results were consistent with the monosaccharide composition analysis and FT-IR spectrum of WGP.

### Water-Soluble Ginseng Polysaccharides Induce C4 Expression in Hepatocytes

**FIGURE 3:**
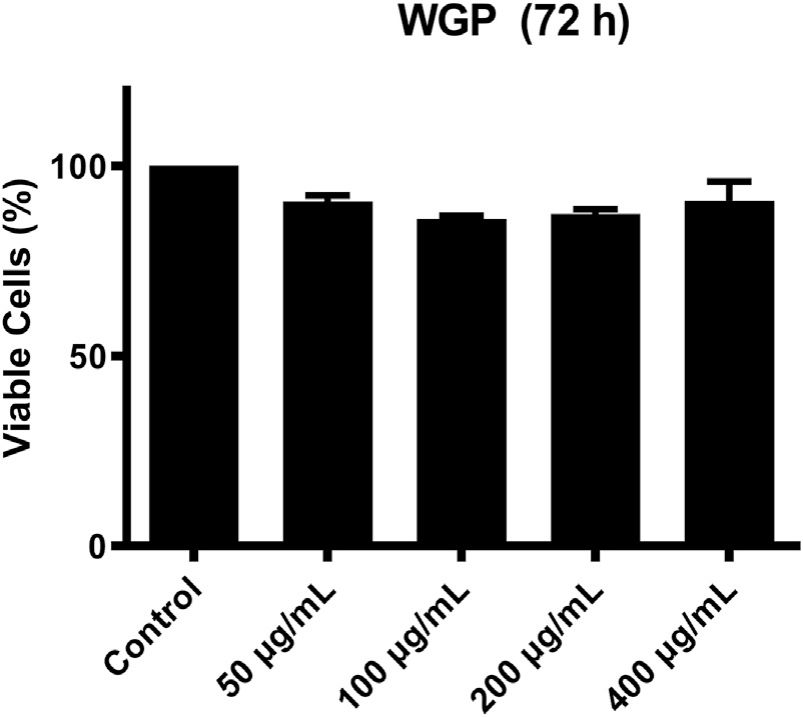
WGP have minimal effect on the growth of hepatocytes. L-O2 cells were treated with WGP at a series of equal ratio gradient concentrations for 72 h and then subjected to MTT assay. Data are presented as mean ± standard errors of the mean (SEM) from 3 independent experiments.

**FIGURE 4:**
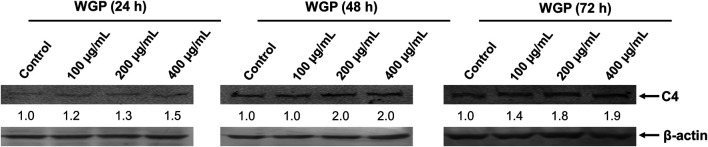
WGP increase C4 production in hepatocytes. L-O2 cells were treated with WGP at a series of equal ratio gradient concentrations for 24, 48 or 72 h. Protein levels of C4 were determined using western blotting. The fold changes of C4 densitometry measurements were compared to β-actin and then normalized to the vehicle control.

C4 is mostly synthesized in the liver (Galibert et al., 1997). Therefore, we chose human normal hepatocyte L-O2 cells as model. First, we investigated the cytotoxic effect of WGP on L-O2 cells by treating the L-O2 cells with WGP for 72 h. The results obtained from MTT assays showed that WGP treatment had minimal effect on viable cells, with the inhibition rate of viable cells less than 14% (Figure 3). Then, we determined the effect of WGP on the protein levels of C4 by western blotting. As shown in Figure 4, WGP increased C4 protein levels as early as 24 h and lasted for 72 h in a dose dependent way. These results show that WGP increase C4 production in hepatocytes.

### Effect of Water-Soluble Ginseng Polysaccharides on C4 Gene Transcription

**FIGURE 5:**
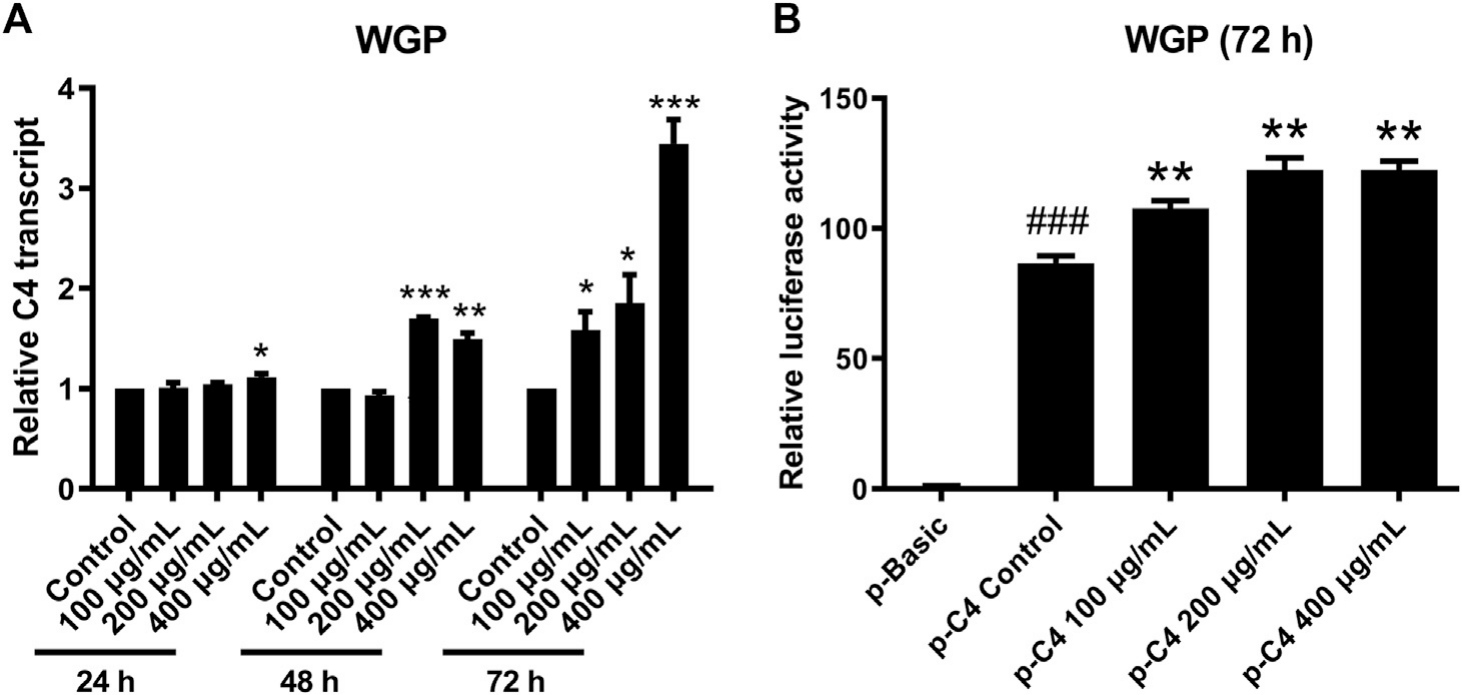
WGP promote C4 gene transcription. L-O2 cells were treated with WGP at a series of equal ratio gradient concentrations for up to 72 h. Real-time PCR method was used to detect the levels of C4 transcripts **(A)**. L-O2 cells was transiently transfected with the full-length pC4 -1,007/+75 (p-C4) plasmid and then treated with WGP or vehicle control for 72 h. Luciferase assays were performed to determine the C4 promoter activity **(B)**. Data are presented as mean of triplicates ±SEM from one representative experiment. * indicates *p* < 0.05, ** indicates *p* < 0.005, and *** indicates *p* < 0.001 compared to vehicle control. ### indicates *p* < 0.001 compared to the basic vector control.

To determine if WGP enhance C4 production through transcriptional mechanisms, we treated L-O2 cells with WGP for 24, 48, and 72 h, and then measured *C4* mRNA levels by real-time PCR. Treatment of L-O2 cells with WGP for 48 and 72 h significantly increased levels of *C4* mRNA, indicating WGP enhances *C4* gene transcription (Figure 5A). To determine if WGP truly enhances *C4* gene transcription, pGL4.19 reporter plasmid consisting of −1,007 to +44 region of the C4 promoter (designated FL) was used to confirm the effect of WGP on *C4* transcription. Transient transfection of pC4-1,007/+44 into L-O2 cells was performed first, then the cells were treated with WGP for 72 h. The p-C4-1,007/+44 construct showed significant reporter activity compared to pGL4.19 basic in the absence or presence of WGP. Interestingly, treatment of the cells with WGP for 72 h significantly enhanced the C4 promoter reporter gene activity (Figure 5B). Taken together, these results demonstrate that WGP increase the production of C4 by promoting *C4* gene transcription.

### Water-Soluble Ginseng Polysaccharides Enhance C4 Transcription Potentially *via* the E-box1 and Sp1 Elements

**FIGURE 6:**
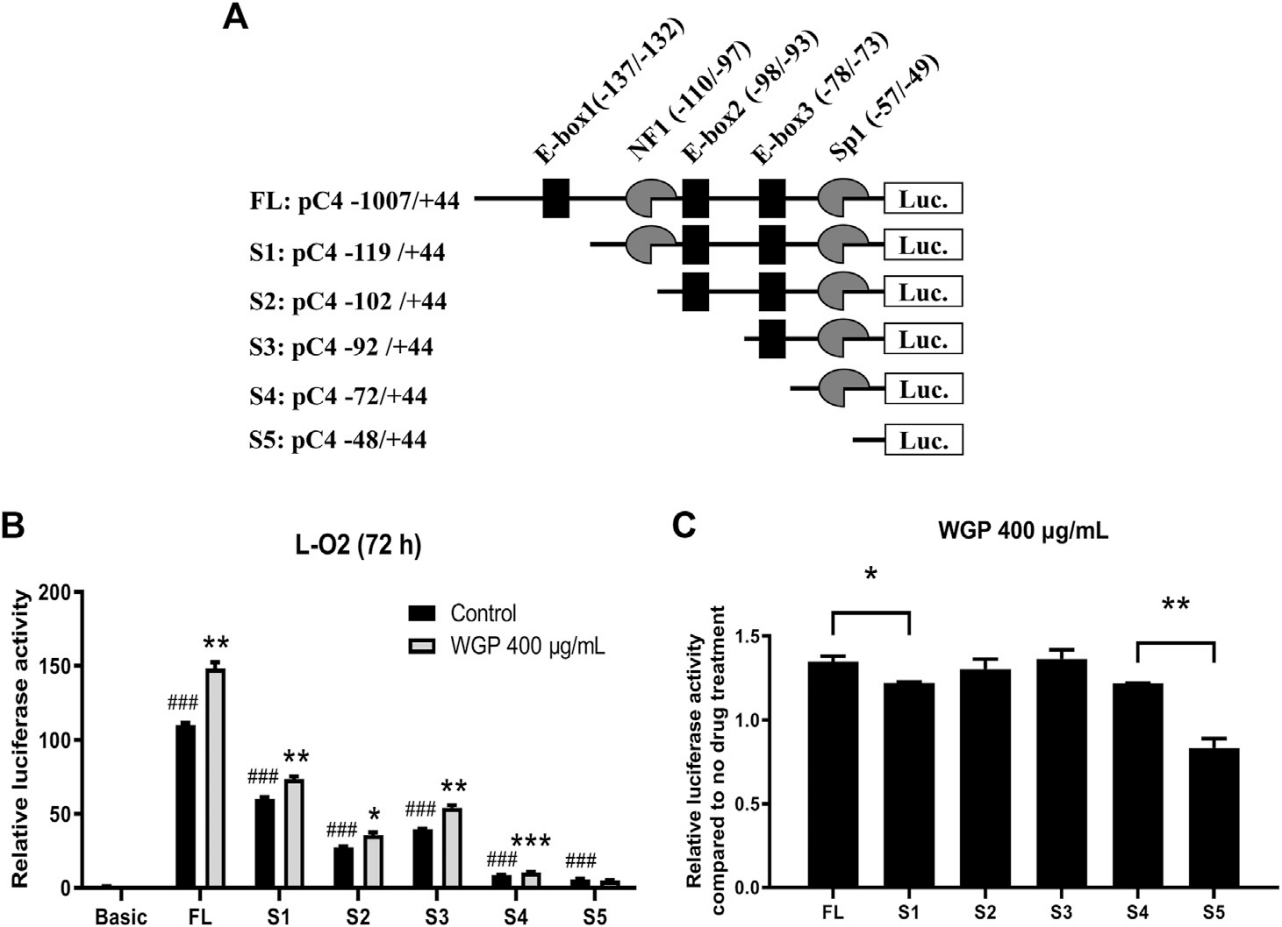
WGP enhance C4 transcription via the E-box1 and Sp1 elements. **(A)** The critical cis-regulatory elements (NF-1, E-box1∼3, Sp1) on the plus (+) and minus (-) DNA strand are shown. Numbering is relative to the C4 translation start site. A series of 5’-deletion constructs were generated by PCR amplification and subcloning into the pGL4.19 basic vector. **(B)** The C4 promoter constructs were transiently transfected into L-O2 cells. Luciferase assay was used to measure the activity of C4 promoter. * indicates *p* < 0.05, ** indicates *p* < 0.005, and *** indicates *p* < 0.001 compared to no drug treatment control. ### indicates *p* < 0.001 compared to basic vector control. **(C)** Fold changes of WGP treatment compared to vehicle control are graphed. * indicates *p* < 0.05 and ** indicates *p* < 0.005. Data are presented as mean of triplicates ±SEM from one representative experiment.

*C4* promoter contains three E-boxes (positions −137 to −132, −98 to −93, and −78 to −73; designated E-box1, E-box2, and E-box3), one NF1 binding site (positions −110 to −97), and one Sp1 (positions −57 to −49) binding element (Figure 6A). To determine which Cis-acting element(s) is (are) vital to the enhancing effect of WGP, a series of 5’ deletion constructs (designated S1-S5; Figure 6A) were generated by using the −1,007 to +144 region of the *C4* promoter as the template. As shown in Figures 6B,C, deletion from −1,007 to −119 significantly decreased *C4* promoter activity and significantly decreased the enhancing effect of WGP on *C4* promoter activity compared to the FL construct, indicating the E-box1 element in this region plays an important role in mediating the enhancing effect of WGP on *C4* promoter. In contrast, further deletions from −119 to −102, −102 to −92, and −92 to −72 did not significantly affect the enhancing effect of WGP on *C4* promoter. Interestingly, deletion from −72 to −48 completely abolished the enhancing effect of WGP, indicating the Sp1 element in this region also plays an important role in mediating the WGP effect on C4 promoter. Taken together, results from our deletion analyses suggest that the E-box1 and Sp1 cis-elements play vital roles in mediating the enhancing effect of WGP on *C4* gene transcription.

## Discussion

Immunomodulatory activity is one of the main functions of ginseng polysaccharides (Guo et al., 2021). The immune system orchestrated by immune organs, immune cells, and immunoreactive substances has the functions of surveillance, defense, and regulation. In the modern world, increasing stress has shifted humans’ lifestyle leading to various health issues, such as cardiovascular disorders, hypertension, diabetes, and hypoimmunity (Parkin and Cohen, 2001). Hypoimmunity predisposes people to infection and cancer. In this study, we explored the immunomodulatory effect of ginseng polysaccharides from the perspective of C4, an important component of the complement system. Here, we demonstrate that WGP possess a strong and significant inductive effect on both protein and mRNA levels of C4 in hepatocyte. Complement deficiency including C4 has been associated with susceptibility to infection. C4 has been shown to inhibit adenoviral infections by inactivating viral capsids (Schröder-Braunstein and Kirschfink, 2019). Reduced levels of complement system components can cause autoimmune diseases due to the lacking of clearance of immunocomplexes (Conigliaro et al., 2019). In these scenarios, taking ginseng polysaccharide or ginseng extract is a potential adjuvant therapy.

C4 is composed of two isotypes, C4A and C4B. Both of them are encoded 10 kb apart in the major histocompatibility complex (MHC) class III area on the sixth human chromosome. *C4A* and *C4B* genes have similarly sequences located separately and closely (Carroll et al., 1984a; Carroll et al., 1984b). *C4* promoter lacks a canonical TATA-box which is commonly found at promoters of genes transcribed by RNA polymerase II (Roeder, 1991). *C4* promoter contains a nuclear factor 1 (NF-1) site at −110 to −97, an Sp1 site at −57 to −49, and three basic helix-loop-helix -like transcription factor sites at −137 to −132, −98 to −93, and −78 to −73, respectively referred to as E-box1, E-box2, and E-box3. These regions appear to be critical for the transcript activity of *C4* gene (Vaishnaw et al., 1998). Thus, we performed deletion analyses within the *C4* gene promoter. We found that E-box1 and Sp1 elements play key roles in WGP-regulated *C4* transcription. A previous study has shown that the E-box motif of *C4* promoter can be recognized by one of the many basic helix-loop-helix (bHLH) or basic helix-loop-helix-leucine zipper (bHLH-LZ) transcription factor family members (Littlewood and Evan, 1995). Moreover, IFN-γ could increase transcription of *C4 via* the E-box sequence at −78 to −73 (Banerjee et al., 2011). Thus, additional studies are warranted to determine the factors modulated by WGP in hepatocytes. However, these studies are not in the scope of this paper.

In conclusion, our results demonstrate that polysaccharides derived from ginseng can significantly increase production of C4 through transcriptional mechanisms. These results provide a molecular explanation for the immunity enhancing function of ginseng extracts.

## Data Availability

The original contributions presented in the study are included in the article/Supplementary Material, further inquiries can be directed to the corresponding authors.
